# Long-Term endocrine outcomes with special emphasis on the gonadal impact of acute lymphoblastic leukemia treatment in females

**DOI:** 10.1007/s00277-026-06783-x

**Published:** 2026-01-14

**Authors:** Hasan Karakaş, Gürkan Tarçın, Elvan Bayramoğlu, Hande Turan, Suheyla Ocak, Volkan Turan, Olcay Evliyaoğlu, Tiraje Tiraje, Hilmi Apak, Oya Ercan

**Affiliations:** 1 Department of Pediatric Endocrinology, Cerrahpaşa Faculty of Medicine, Istanbul University-Cerrahpaşa, Istanbul, Türkiye; 2 Department of Pediatric Endocrinology, Kocaeli University Faculty of Medicine, Kocaeli, Türkiye; 3 Department of Pediatric Hematology-Oncology, Cerrahpaşa Faculty of Medicine, Istanbul University-Cerrahpaşa, Istanbul, Türkiye; 4 Department of Obstetrics and Gynecology, Istanbul Health and Technology University School of Medicine, Istanbul, Türkiye

**Keywords:** Acute lymphoblastic leukemia, Anti-Müllerian hormone, Endocrine disorders, Fertility, Gonadal toxicity, Preservation

## Abstract

This study aims to explore the long-term endocrine and gonadal effects of chemotherapy and radiotherapy in female acute lymphoblastic leukemia (ALL) patients. A cohort study included girls diagnosed with ALL and treated between 2000 and 2020. Patients with at least 2 years elapsed since treatment completion were included. Endocrinological evaluations included anthropometric measures and pubertal status, as well as fasting insulin, glucose, lipid levels, and hormone assessments for adrenal, and thyroid functions. Reproductive functions were evaluated based on gonadotropin, estradiol, and anti-Müllerian hormone (AMH) levels. A total of 51 female patients were included. At the time of study participation, the mean age was 14.7 years, and the mean time since treatment completion was 9.4 years. At least one endocrine disorder was present in 39.2% of participants, with dyslipidemia, insulin resistance, and obesity being the most common. Low AMH levels (< 1.1 ng/dL) were found in 41.6%, particularly in those who underwent bone marrow transplantation. A significant positive correlation was found between the time elapsed since treatment and AMH levels (*p* < 0.001, *r* = 0.612), while age at diagnosis, risk group (standard, intermediate or high risk), and cranial radiotherapy showed no significant associations. A substantial proportion of ALL survivors developed endocrine complications, with ovarian reserve compromised in over 40% of cases. Notably, this is the first cohort study to demonstrate a significant positive correlation between AMH levels and the time elapsed since treatment, suggesting a potential for gonadal recovery except in those exposed to intensive chemotherapy or transplantation.

## Introduction

The unfavorable effects of chemotherapeutic drugs and radiotherapy used in the treatment of acute lymphoblastic leukemia (ALL) on the endocrine system may manifest both in the short term and t he long term. Despite the rising survival rates due to the modification of treatment protocols and the advancement of supportive care over the years, patients with ALL still require prolonged monitoring for endocrine disorders. As the likelihood of encountering long-term treatment-related complications also increases [1, 2].

The severity of long-term toxicity can vary among the risk groups due to differences in the drugs used in chemotherapy, their dosages, and the utilization of cranial radiotherapy [3]. Notably, more intense chemotherapy regimens, particularly those used in cases of relapse or bone marrow transplantation, are associated with more severe endocrine side effects [4].

In leukemia treatment, cyclophosphamide presents a high risk for gonadal toxicity, while doxorubicin has a moderate risk, and methotrexate, vincristine, and mercaptopurine are associated with lower risks [5]. Neoplastic diseases and treatments, particularly those involving higher doses of alkylating agents and radiation targeting the pelvic/gonadal area or the central nervous system, can compromise gonadal functions. This can lead to ovarian insufficiency and infertility, primarily through a reduction in oocyte count [3, 6].

Most studies on gonadotoxicity following cancer treatment have concentrated on aspects like menstrual cycle regularity, the occurrence of pregnancy, and gonadotropin levels [3]. However, even when gonadotropin levels and ovarian hormonal activity remain intact post-treatment, ovarian reserve may still be diminished. Therefore, anti-Müllerian hormone (AMH) serves as a critical marker for assessing ovarian reserve. The aim of this study is to examine the long-term endocrine effects, particularly focusing on the impact on gonadal function through AMH levels, in female cases treated for ALL. Additionally, we aim to reveal the relationship between the observed effects and treatment toxicity.

## Materials and methods

### Study design and selection of participants

This cohort study was conducted on girls diagnosed with ALL and treated between 2000 and 2020. The patients who completed the treatment and had at least 2 years elapsed since treatment were identified from the medical records and formally invited to participate in the study. Those who could be reached and agreed to participate were included in the study.

Risk classification and treatment procedures, including details regarding radiotherapy, chemotherapy, and bone marrow transplantation, were obtained from the medical records of the patients.

### Treatment protocol

All patients were treated within the backbone of BFM-based protocols that share a comparable four-phase structure: induction (Protocol IA/IB), consolidation (Protocol M for standard/intermediate risk; HR-1’, HR-2’, HR-3’ blocks for high risk), re-induction (Protocol II), and maintenance.

Between 2000 and 2009, patients were treated according to the modified ALL-BFM 95 protocol [7], and from 2009 to 2020, according to the ALL-IC BFM 2009 protocol [8]. Both protocols maintained identical chemotherapy phases and cumulative dose ranges for key agents, differing mainly in risk stratification criteria and indications for cranial radiotherapy.

The cumulative dose during induction therapy corresponded to approximately 60 mg/m²/day of prednisone for 21 days (total ≈ 1,260 mg/m²), vincristine 6 mg/m² (4 doses × 1.5 mg/m²), daunorubicin 60–120 mg/m² depending on risk group, and L-asparaginase 30,000–80,000 U/m² (6–8 doses × 5,000–10,000 U/m²). Consolidation included high-dose methotrexate (1–5 g/m² × 4 courses) and cyclophosphamide (3–4 g/m² total). For patients classified as high-risk, additional therapy blocks incorporated high-dose cytarabine (total cumulative dose of 4–8 g/m²) and dexamethasone (total cumulative dose of approximately 100 mg/m² administered as 20 mg/m²/day for 5 days).

Cranial radiotherapy was given only according to protocol-specific criteria: in modified BFM 95 protocol, for Central nervous system (CNS) involvement (CNS3), t(4;11) translocation, T-cell phenotype, poor prednisone response, or high minimal residual disease (≥ 5 × 10⁻⁴ at week 12); in BFM 2009, for T-ALL with initial WBC > 100 000/µL, CNS3 disease, or non-transplanted high-risk patients.

### Endocrinological evaluation

All patients’ height and weight were assessed according to Turkish children’s growth charts, and their body mass index (BMI) percentiles were calculated [9]. Short stature was defined as a height below the 3rd percentile [10]. Obesity in children was identified with a BMI percentile above the 95th, while in adults, it was defined as a BMI greater than 30 kg/m² [11]. Overnight fasting insulin, glucose, and lipid levels were measured, and insulin resistance was calculated using the homeostasis model assessment (HOMA) index, with the threshold for insulin resistance set at > 2.2 for prepubertal and > 3.8 for pubertal girls [12]. For adults, a fasting serum insulin level above 15 µU/mL was considered indicative of insulin resistance [13]. High lipid levels were defined as total cholesterol > 200 mg/dL, low-density lipoprotein cholesterol (LDL) > 130 mg/dL, and triglycerides > 150 mg/dL, with any elevation in one of these parameters considered as dyslipidemia [14].

The presence of adrenal insufficiency was assessed using morning cortisol levels. According to this, morning cortisol levels above 15 were considered normal, while individuals with cortisol levels below 15 ng/dL on two occasions underwent low-dose ACTH testing, and a peak cortisol level below 18 was determined as inadequate [15]. Thyroid gland disorders were evaluated through TSH and fT4 levels.

### Evaluation of reproductive functions

Patients’ pubertal status was assessed according to the Tanner stages. Reproductive functions were evaluated based on follicle-stimulating hormone (FSH), luteinizing hormone (LH), estradiol, and AMH levels measured using with electrochemiluminescence method “ECLIA” (Roche Diagnostics™, Elecsys, Cobas E 801, Mannheim, Germany). In cases of the absence of thelarche beyond 13 years of age, regression in puberty, or amenorrhea in previously pubertal girls, testing for gonadotropin and estradiol levels was prompted. Accordingly, a diagnosis of hypergonadotropic hypogonadism was made in the presence of high follicle-stimulating hormone (FSH) levels (> 20 mIU/mL) and low estradiol levels, while a diagnosis of hypogonadotropic hypogonadism was established when both gonadotropin and estradiol levels were undetectably low. The optimal threshold values for anti-Müllerian hormone (AMH) in clinical practice to indicate decreased ovarian reserve have been reported as ranging between 0.5 and 1.1 ng/ml [16, 17]. Accordingly, in our study, AMH levels below 1.1 ng/ml were considered indicative of decreased ovarian reserve.

### Statistical analyses

The Statistical Package for Social Sciences version 27 (Chicago, Illinois, USA) was used for statistical analyses. Histograms, Q-Q plots, SD-to-mean ratio, kurtosis and skewness were used to assess the data distribution. Data with parametric distribution was expressed as mean ± SD, and data with nonparametric distribution was expressed as median (minimum-maximum). Spearman correlation analysis was used to determine the relation between elapsed time since treatment and AMH levels. A linear regression analysis was conducted to identify the predicting factor on AMH levels. Statistical significance was set at *p* < 0.05.

## Results

A total of 147 female patients were identified. Eleven were deceased, 59 were unreachable by phone, and 26 of the 77 who were successfully contacted declined participation. The study was completed with 51 participants who provided consent and underwent evaluation.

At the time of study participation, the mean age of the participants was 14.7 ± 5.8 years (3.3–29.1). The mean age at diagnosis was 5.4 ± 3.9 years (0.6–17.1), and the mean time elapsed since treatment completion was 9.4 ± 5.3 (2–22.4) years. 7 girls (13.7%) were pubertal during the period of chemotherapy. Of the patients, 16 girls (31.3%) were classified as standard risk (SR), 26 girls (50.9%) as intermediate risk (IR), and 9 girls (17.6%) as high risk (HR). Seven patients (13.7%) had cranial radiotherapy (six were treated with 12 Gy and one with 18 Gy). None of the patients had recurrence of their disease during the time elapsed since treatment completion, and among the high-risk group, three patients underwent bone marrow transplantation.

At least one endocrine disorder was present in 20 girls (39.2%), with the most common disorders being dyslipidemia in 9 girls (17.6%), insulin resistance in 8 girls (15.6%), and obesity in 4 girls (7.8%), while none of the patients had adrenal insufficiency or hypogonadotropic hypogonadism (Table 1). All three transplant recipients exhibited at least one endocrinological disorder, and dyslipidemia was detected in all of them. One patient from the standard risk group developed precocious puberty at the age of 7 and was started on GnRH analog treatment.

**Table 1:** Distribution of endocrinological disorders in the study group

Disorder	Total (*n* = 51)	Standard risk (*n* = 16)	Intermediate risk (*n* = 26)	High risk (*n* = 9)
Insulin resistance	8 (15.6%)	2 (12.5%)	4 (15.3%)	2 (22.2%)
Dyslipidemia	10 (19.6%)	0	6 (23%)	4 (44.4%)
Obesity	4 (7.8%)	0	4 (15.3%)	0
Short Stature	3 (5.8%)	0	2 (7.6%)	1 (11.1%)
Hypergonadotropic hypogonadism	2 (3.9%)	0	1 (3.8%)	1 (11.1%)
Subclinical hypothyroidism	2 (3.9%)	0	1 (3.8%)	1 (11.1%)
Precocious puberty	1 (1.9%)	1 (6.3%)	0	0
Central hypothyroidism	0	-	-	-
Adrenal insufficiency	0	-	-	-
Hypogonadotropic hypogonadism	0	-	-	-
**Patients with ≥ 1 disorder**	**20 (39.2)**			

Results are presented as counts (n) and percentages (%). Prevalence rates were calculated for each disorder within the study group. No cases were observed for adrenal insufficiency or hypogonadotropic hypogonadism

### Ovarian functions and associated factors

As for reproductive functions, AMH levels were assessed only in 48 patients who were pubertal at the time of the study. A total of 20 patients (41.6%) had low AMH levels (< 1.1 ng/dL). Among the standard-risk group (*n* = 15), 5 patients (33.3%) had low AMH levels; among the intermediate-risk group (*n* = 24), 9 patients (37.5%) showed low AMH levels. In the high-risk group (*n* = 9), all three patients who underwent bone marrow transplantation had undetectably low AMH levels (< 0.01 ng/dL). while 3 of the remaining 6 patients (50%) had decreased AMH levels. However, the proportion of patients with low AMH levels did not differ significantly among the risk groups (*p* = 0.720).

**Fig. 1:**
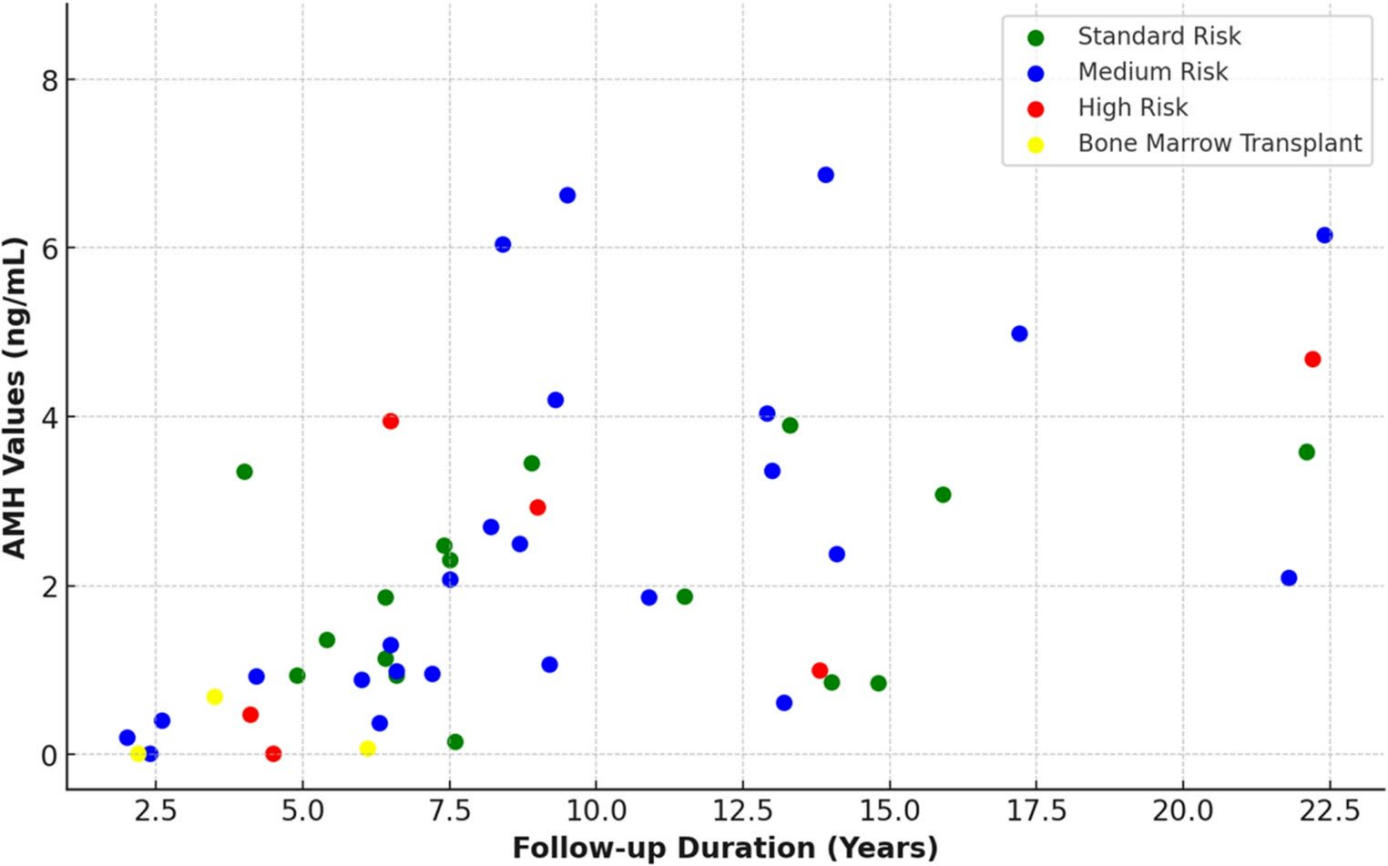
Scatter plot of anti-Müllerian hormone levels and time elapsed since treatment, stratified by risk classification

A multiple linear regression analysis was conducted to determine the factors influencing AMH levels. Multiple linear regression analysis showed that age at diagnosis and risk group did not significantly influence AMH levels; however, the time elapsed since treatment completion had a significant positive effect. In other words, regardless of the age at diagnosis, risk group, or pubertal status at the time of treatment, a longer time since treatment was associated with higher AMH levels (Table 2). Also, a strong correlation was obtained between AMH levels and time elapsed since treatment (*p* < 0,001, *r* = 0.612) (Fig. 1). In the analysis excluding the three patients who underwent bone marrow transplantation, the remaining pubertal patients were categorized according to the time elapsed since treatment completion (< 5 years, 5–15 years, and > 15 years). The proportion of patients with low AMH levels differed significantly among these three groups (*p* < 0.001). Within the first 5 years after treatment, all but one patient had low AMH levels, whereas no patient had low AMH levels more than 15 years after treatment.

**Table 2:** Linear regression model for the factors affecting anti-Müllerian hormone levels in pubertal females treated for acute lymphoblastic leukemia

	Unstandardized coefficients	Sig.	95% confidence interval for B	
	B	*p*	Lower bound	Upper bound
Age at diagnosis	0.033	0.776	−0.198	0.263
Elapsed time since treatment	0.191	< 0.001	0.088	0.294
Risk group	0.008	0.984	−0.804	0.820
Pubertal status	−0.114	0.927	−2.588	2.360

Results are displayed as unstandardized coefficients (B), with associated p-values and 95% confidence intervals. Boldface font indicates statistically significant values (*p* ≤ 0.05). The analysis identifies the elapsed time since treatment as a significant factor positively affecting AMH levels, while other factors, such as age at diagnosis, risk group, and pubertal status, were not significant

Hypergonadotropic hypogonadism was detected in 2 out of the 3 patients with undetectably low AMH levels who had undergone intense chemotherapy due to bone marrow transplantation. Apart from these 2 patients, all other participants exhibited normal pubertal development, as well as normal gonadotropin and estradiol levels according to their pubertal stages.

## Discussion

In this study, long-term post-treatment observation revealed that more than one-third of the surviving girls who were treated for ALL in childhood developed at least one endocrinological disorder. In the literature, the rate of endocrine disorders following ALL treatment has been reported to be similar to the general prevalence range in childhood cancer survivors ranging between 47.6% and 59.5% [2].

In our study, the most frequently observed endocrinological effects were identified as dyslipidemia, insulin resistance, and obesity, which are in fact interrelated endocrine and metabolic disorders. It is well-known that patients who have received cumulative high doses of steroids or radiotherapy during treatment are at risk of developing obesity and dyslipidemia [18]. While it is also well-known that steroids can increase insulin resistance and enhance lipid accumulation [19], the hypothalamic damage caused by cranial radiotherapy is proposed to lead to leptin insensitivity and predispose individuals to obesity [20]. However, it was also shown that obesity rates increased after treatment, regardless of whether patients received cranial radiotherapy or not, compared to their pre-treatment status [18].

Additionally, patients adopting a sedentary lifestyle during treatment and parents showing tolerance towards unhealthy diets are also reasons for the increase in obesity rates [21]. However, the frequency of obesity observed in our cohort during the time elapsed since treatment was comparable to that reported in healthy young adults [22]. This suggests that the tendency towards obesity observed during treatment does not increase over the long term compared to the healthy population.

As the success of current ALL treatment protocols and, consequently, survival rates have increased, concerns regarding fertility have also intensified. Regarding gonadal effects, although treatment for ALL in childhood is considered to be less harmful to the gonads compared to other solid cancer protocols that include high doses of cyclophosphamide and radiation [23], studies focusing on gonadal effects in ALL survivors are limited, and results are presented alongside other types of cancer, taking into account the effects of total doses of alkylating agents or radiation [6]. Since chemotherapy is administered as a multi-drug regimen, determining the individual relative contribution of each drug to gonadal impact is challenging. However, it is widely recognized that cyclophosphamide poses a significant risk to gonadal health [5]. Although the effects of cyclophosphamide are thought to be dose-dependent, a critical dose that would definitively predict future fertility outcomes has not been established [3]. In our study, the absence of significant differences in AMH levels across the SR, IR, and HR groups may suggest that even relatively low cumulative doses of cyclophosphamide, when used in combination with other agents, could contribute to decreased ovarian reserve.

Although it is mentioned that gonadal impact is less following cancer treatment given before puberty, our study found no difference in AMH levels between girls treated before puberty and those treated during adolescence. There are studies reporting that ovaries, which are functionally inactive before puberty and exposed to alkylating agents, suffer less damage compared to patients in puberty [24]. Even though there are studies recommending the use of GnRH analogs for puberty blockade in selected cases to minimize ovarian impact during chemotherapy [25], the mechanisms explaining how the ovary is protected in its prepubertal state have not yet been clearly demonstrated [26].

The most exciting finding of our study is the relationship between the time elapsed since chemotherapy and AMH levels, which, although the study is not longitudinal, suggests gonadal recovery over time. The first longitudinal study on ovarian reserve following childhood cancer treatment examined the AMH levels before and after treatment in a heterogenous group with 22 patients diagnosed with different types of cancer [3]. The patients were classified into standard, intermediate, and high risk based on the chemotherapy intensity and radiotherapy regimens. Accordingly, while AMH levels remained undetectable in the high-risk group, girls in the standard and intermediate risk groups showed recovery to levels similar to those before treatment. Similarly, a study from Japan involving three patients diagnosed with myelodysplastic syndrome (MDS), acute myeloid leukemia (AML), and ALL reported that, unlike the patients diagnosed with AML and MDS who underwent bone marrow transplantation and had undetectably low AMH levels, an increase in AMH level was observed in the patient diagnosed with ALL. This recovery has been attributed to the resumption of the normal pattern of early follicle growth in the ovaries. In contrast, the lack of recovery in high-risk groups has been attributed to a profound loss of the primitive follicle pool, which is incapable of producing growing follicles to secrete AMH [27]. In a cohort consisting only of patients diagnosed with ALL, a positive relationship between the time elapsed since treatment and AMH levels was observed, similar to our study. Accordingly, it was shown that women who were within 10 years of completing treatment had lower AMH levels compared to those in whom more than 10 years had passed since treatment completion [28]. On the other hand, it was reported that about 90% of women undergoing Hematopoietic Stem Cell Transplantation (HSCT) had severe gonadal toxicity and permanent infertility [4]. In our study, all three patients who had undergone bone marrow transplantation showed undetectably low AMH levels, and additionally, two of them had developed hypergonadotropic hypogonadism. This suggests that the intensive chemotherapy involved in bone marrow transplantation causes serious and likely irreversible damage to the ovarian follicle pool. It should also be noted that low AMH levels do not necessarily indicate irreversible ovarian failure or preclude the possibility of pregnancy, as partial recovery and spontaneous conception may still occur over time.

In survivors of childhood cancer, cranial radiotherapy administered during treatment can lead to hypogonadotropic hypogonadism in later periods due to disruption of hypothalamus-pituitary function, with the risk depending on the administered dose. In a large cohort study, although it was shown that exceeding a radiation dose of 22 Gy adversely affects fertility in later periods [29], gonadotropin deficiency is most commonly reported following a radiation dose of greater than 40 Gy [30]. In our study, although the number of patients who received radiotherapy was limited, all were administered cranial radiotherapy at low doses (12–18 Gy), which did not result in any negative central effects. This suggests that low doses may be safer for the hypothalamo-pituitary-gonadal axis.

The study is subject to certain limitations. Notably, the observed positive correlation between the elapsed time since treatment and the AMH levels is derived from cross-sectional data rather than from longitudinal analysis. To more clearly demonstrate this finding, longitudinal studies including detailed follicle assessments via pelvic ultrasound are warranted. Another limitation is the small number of patients who underwent bone marrow transplantation, which limits the generalizability of our findings regarding the impact of intensive chemotherapy on ovarian reserve.

## Conclusion

The results of this study show that obesity and related metabolic complications were the most common long-term endocrine disorders observed. Additionally, this is the first study in a cohort setting to demonstrate that ovarian reserve may improve over time following chemotherapy, except in patients who underwent bone marrow transplantation or intensive chemotherapy for relapse. Longitudinal studies with larger cohorts are needed to further clarify factors affecting gonadal impairment and recovery, aiding in identifying candidates for fertility preservation or expectant management.

## Data Availability

The data that support the findings of this study are available from the corresponding author upon reasonable request.

## Declarations

### Reporting guideline

This study was reported in accordance with the STROBE (Strengthening the Reporting of Observational Studies in Epidemiology) statement.
